# A comprehensive characterization of chronic norovirus infection in immunodeficient hosts

**DOI:** 10.1016/j.jaci.2019.07.036

**Published:** 2019-11

**Authors:** Li-An K. Brown, Christopher Ruis, Ian Clark, Sunando Roy, Julianne R. Brown, Adriana S. Albuquerque, Smita Y. Patel, Joanne Miller, Mohammed Yousuf Karim, Samir Dervisevic, Jennifer Moore, Charlotte A. Williams, Juliana Cudini, Fernando Moreira, Penny Neild, Suranjith L. Seneviratne, Sarita Workman, Christos Toumpanakis, Claire Atkinson, Siobhan O. Burns, Judith Breuer, David M. Lowe

**Affiliations:** a Department of Infectious Diseases, Royal Free London NHS Foundation Trust, London, United Kingdom; b Department of Microbiology, Whittington Health NHS Trust, London, United Kingdom; c Division of Infection and Immunity, University College London, London, United Kingdom; d Molecular Immunity Unit, Department of Medicine, University of Cambridge, Medical Research Council (MRC)–Laboratory of Molecular Biology, Cambridge, United Kingdom; e Department of Histopathology, Royal Free London NHS Foundation Trust, London, United Kingdom; f Department of Microbiology, Virology and Infection Prevention and Control, Great Ormond Street Hospital for Children NHS Foundation Trust, London, United Kingdom; g Institute of Immunity and Transplantation, University College London, Royal Free Campus, London, United Kingdom; h Oxford University Hospitals NHS Trust and NIHR Oxford Biomedical Research Centre, Oxford, United Kingdom; i Royal Surrey County Hospital NHS Foundation Trust, Egerton Road, Guildford, Surrey, United Kingdom; j Pathology, Sidra Medicine, Doha, Qatar; k Norfolk and Norwich University Hospital, Norwich, Norfolk, United Kingdom; l Department of Clinical Immunology, Royal Free London NHS Foundation Trust, London, United Kingdom; m St George's University Hospitals NHS Foundation Trust, London, United Kingdom; n Department of Gastroenterology, Royal Free London NHS Foundation Trust, London, United Kingdom

To the Editor:

Chronic norovirus infection (CNI) can be devastating in patients with immunodeficiency.^1^ We sought to investigate immunologic and virologic factors associated with chronic infection and document outcomes from a structured treatment algorithm.

We identified 10 patients with CNI (1 referred from Oxford University Hospitals), which was defined as compatible symptoms (diarrhea, weight loss, and vomiting) or malabsorption with stool samples with positive PCR results for norovirus for more than 3 months. We also recruited control groups (see the Methods section in this article's Online Repository at www.jacionline.org).

Nine patients had CVID, and 1 had chronic lymphocytic leukemia and secondary antibody deficiency. Comorbidities and concurrent infections are detailed in Table E1 in this article's Online Repository at www.jacionline.org. All patients had diarrhea, 3 had significant vomiting, and most had weight loss. The majority had low vitamin E, folate, or B12 levels, and patients A and H had low zinc levels; therefore all 10 patients had evidence of malabsorption. Three required parenteral nutrition.

In comparison with other patients with CVID from our center, those with CNI (n = 8) had lower long-term average CD3^+^ (median, 0.61 vs 1.12 × 10^9^/L; *P* = .009) and CD19^+^ (median, 0.07 vs 0.18 × 10^9^/L; *P* = .017) cell counts (Fig 1). From the earliest available data, 6 patients with CNI were classified as Freiburg group 1A, and 3 were classified as Freiburg group 1B (all of whom eventually met the criteria for group 1A because of a subsequent increase in CD21^−^ B-cell counts). T-cell proliferation with 8 μg/mL PHA was impaired in all patients (median, 8.1% [5.2% in 6 patients undergoing immunosuppression and 9.5% in 4 patients off immunosuppression]; range, 0.5% to 33.7% of contemporaneous control; see Table E4 in this article's Online Repository at www.jacionline.org). In 7 of 8 patients, we observed a possible temporal relationship between decreasing CD3^+^ and CD19^+^ cell counts and diagnosis or earliest date of norovirus acquisition (see Fig E1 in this article's Online Repository at www.jacionline.org). Often this correlated with iatrogenic immunosuppression for inflammatory or autoimmune disease: 2 patients received high-dose corticosteroids, and 1 received corticosteroids and rituximab. The patient from Oxford University Hospitals was receiving prednisolone and mycophenolate mofetil at diagnosis. Four patients (D, E, I, and J) received corticosteroids with or without infliximab for “CVID enteropathy” before norovirus diagnosis.

**Fig 1:**
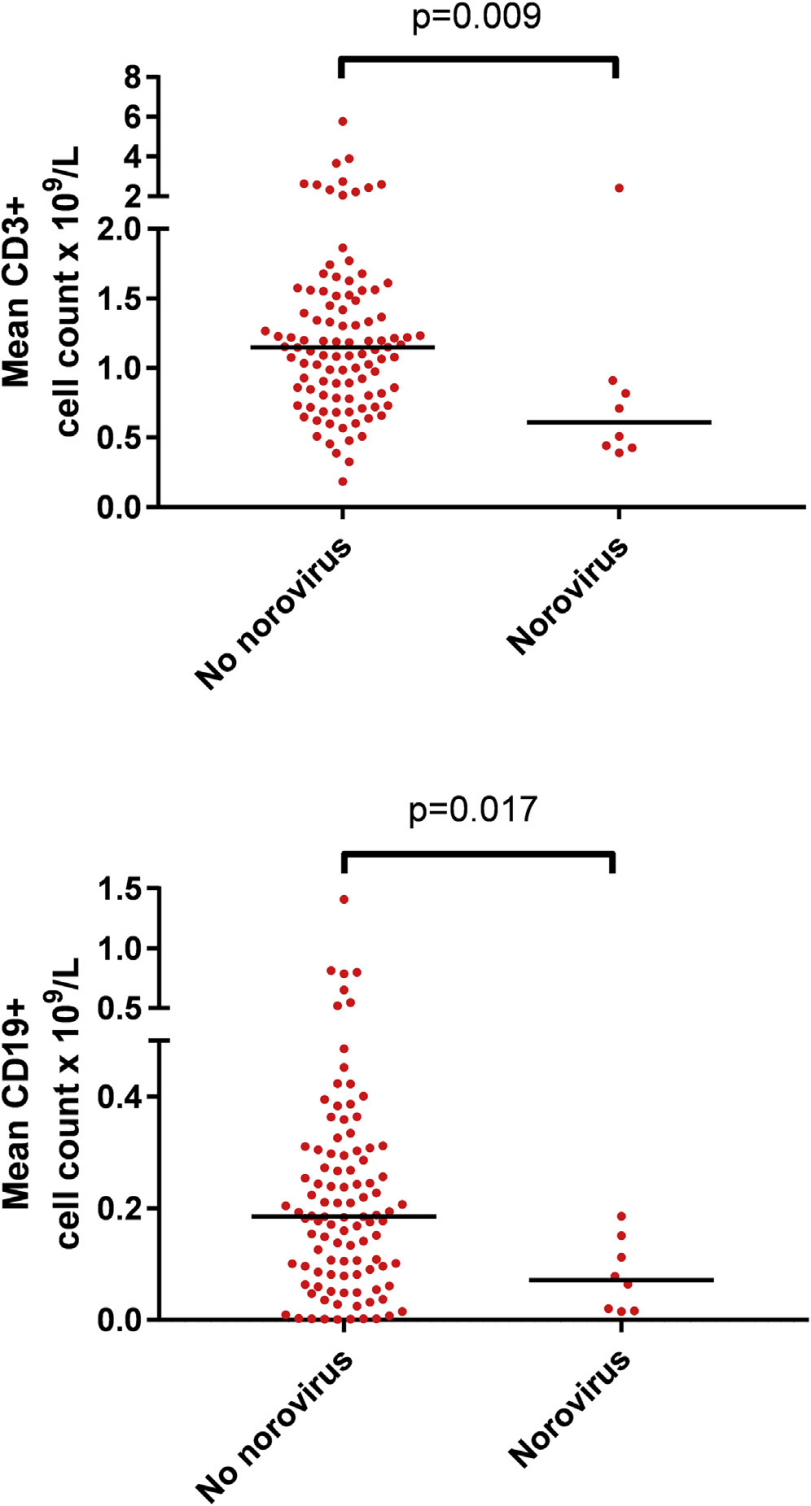
Long-term average (from 2000 [or date of CVID diagnosis, if later] to 2018) CD3^+^ and CD19^+^ values for 8 patients with CVID with CNI versus 113 patients with CVID without CNI. *Lines* represent medians. *P* values are from Mann-Whitney tests.

We further characterized peripheral blood lymphocytes in patients with CVID through flow cytometry. Versus healthy control subjects, patients with CNI had significantly reduced CD27^+^ B-cell counts, including switched memory cells and CD80^+^ and CXCR5^+^ B-cell percentages but increased CD21^−^ (of which 68.6% were CXCR5^−^) and transitional B-cell percentages (see Fig E2, *A*, in this article's Online Repository at www.jacionline.org). There was no difference in numbers of plasmablasts and CD5^+^ or CD86^+^ B cells. Patients with CVID without CNI (n = 8) also demonstrated a significant (but smaller) increase in CD21^−^ B-cell percentages versus control subjects but no difference in other populations (see Fig E2, *B*).

Norovirus-infected patients had significantly increased numbers of CD4^+^ follicular helper T (CXCR5^+^PD1^+^) cells (9.5% ± 6.3% of total CD4^+^ T cells) and overall programmed cell death protein 1 (PD-1)^+^CD4^+^ cells (36.7% ± 22.4% of total CD4^+^ T cells), with a trend toward decreased naive (CD45RA^+^CD62L^+^) cell percentages versus control subjects (see Fig E2, *A*). Patients with CVID without norovirus also demonstrated increased follicular helper T-cell counts but not total PD-1^+^CD4^+^ T-cell percentages.

Table E2 in this article's Online Repository at www.jacionline.org summarizes gastrointestinal findings. Duodenal biopsy specimens demonstrated villous atrophy and intraepithelial lymphocytosis in 7 patients (see Fig E3, *A*, in this article's Online Repository at www.jacionline.org); both patients with “normal” duodenal histology had profound small bowel villous atrophy observed endoscopically (see Fig E3, *B*). Therefore all 9 patients with mucosal assessment had macroscopic or histologic evidence of villous atrophy.

Immunohistochemical staining in 6 biopsy specimens revealed increased intraepithelial CD3^+^ lymphocytes, predominantly CD8^+^. CD4^+^ lymphocyte frequencies varied. Patients with CVID had absent or severely reduced numbers of CD19^+^ lymphocytes. All patients had granzyme B–positive cells, with perforin staining in 3: their distribution was identical to CD8^+^ T cells, suggesting that epithelial cell injury is mediated in part by cytotoxic cells.

We performed norovirus whole-genome sequencing^2^ to exclude cross-transmission. Patient sample consensus sequences were used with worldwide surveillance reference strains to construct phylogenetic trees (see Fig E4 in this article's Online Repository at www.jacionline.org). Patients A, B, and E were infected with the GII.4 variant New Orleans 2009, and patients D and J were infected with viruses clustering between GII.4 US95/96 and GII.4 Osaka 2007, but the viruses separated in the trees (see Fig E5 in this article's Online Repository at www.jacionline.org). Other patients were infected with distinct genotypes or variants (see Table E3 in this article's Online Repository at www.jacionline.org), and therefore no evidence supported transmission within the cohort.

We estimated patient-specific infection intervals by calculating divergence and ancestor dates (see Table E3). Patient F was likely infected within the 5 months before diagnosis, but intervals for other patients suggested years of antecedent infection. Sequencing also demonstrated relapses with the same strain after long periods of negative PCR results (up to 11 months in patient E [see below] and 14 months in patient F), which was far longer than has been described previously.^3^

Viral diversity (number of nucleotide differences between reads at a specific site)^4^, ^5^ was extremely high in our cohort compared with that in the control groups (see Fig E6, *A*, in this article's Online Repository at www.jacionline.org). This diversity is rapidly generated, for example, in patient A within 6 months of the most recent common ancestor (see Fig E6, *B*) and then fluctuates over time (see Fig E6, *C*). The diversity within patient C, whose virus had GII.P7 RdRp, was lower than in those with GII.P4 RdRp, which is consistent with a lower mutation rate with the GII.P7 polymerase.^6^ Diversity or estimated infection duration did not correlate with drug treatment outcome, but notably, only patient C has achieved sustained viral clearance.

Dietary interventions were occasionally of benefit (Fig 2); 3 of 8 patients improved symptomatically with a lactose-free diet, and 2 of 10 patients improved symptomatically with a gluten-free diet. High-dose intravenous immunoglobulin (1 g/kg per week for 4 to 6 weeks) had no clear effect. Patient D reported modest symptomatic benefit with Pentaglobin (an immunoglobulin product containing IgA and IgM in addition to IgG; Biotest AG, Dreieich, Germany).

**Fig 2:**
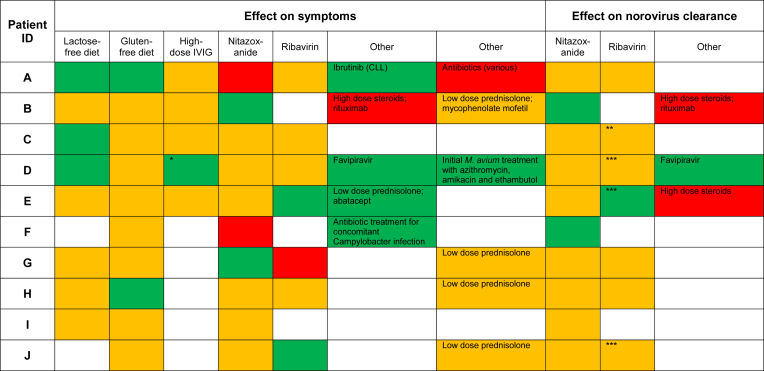
Effect of interventions on symptoms and norovirus clearance. Effect on symptoms: green, improvement; orange, no clear effect; red, deterioration; and white, not attempted. Effect on norovirus clearance: green, sustained decrease in viral load or multiple negative samples; orange, no clear effect; red, relapse or increase in viral load; and white, not attempted. *Symptomatically improved with Pentaglobin but no viral clearance. **Apparent initial response but subsequent relapse during treatment. Further episode of infection 2 years later resolved rapidly with ribavirin therapy. ***Evidence of likely treatment-related anemia.

Nitazoxanide (500 mg administered twice daily for a minimum of 4 weeks) was tried in all patients. Patients B and F appeared to clear norovirus after treatment (with symptomatic improvement in patient B, whereas patient F's symptoms worsened during therapy). However, both later relapsed (after 6 and 14 months, respectively). Patient B restarted nitazoxanide and had negative PCR results for 5 months until relapsing after high-dose steroids and rituximab for hemolytic anemia. Patient G symptomatically improved with nitazoxanide but did not clear norovirus (see Fig E7 in this article's Online Repository at www.jacionline.org), whereas patient A symptomatically deteriorated; other patients saw no effect.

Seven patients received ribavirin therapy (initial dose, 200 mg in the morning/400 mg in the evening and titrated according to serum levels). Patient E saw symptomatic improvement (see Fig E8 in this article's Online Repository at www.jacionline.org) and cleared norovirus from stool. However, discontinuation after 11 months of negative results resulted in rapid relapse and was also associated with inflammatory arthritis. Ribavirin reintroduction (with concomitant corticosteroid therapy for arthritis) did not clear norovirus, and the patient had treatment-related anemia. Patients D and J also had anemia while receiving ribavirin. Patient C had an initial virologic response but relapsed despite continuous therapy. She eventually cleared norovirus spontaneously, 2 months after discontinuing ribavirin. Patient J's symptoms improved with a small viral load decrease before having anemia. Conversely, patient G's symptoms symptomatically deteriorated on ribavirin, whereas other patients saw no clear effect. Patient D was treated experimentally with favipiravir, as we have described.^7^

Despite the negative effect of significant immunosuppression, low-dose prednisolone improved patient E's symptoms (see Fig E8). Other patients receiving low-dose prednisolone have not noted detrimental effects. Patient A with chronic lymphocytic leukemia experienced dramatic symptomatic improvement after receiving ibrutinib to attempt to achieve immune reconstitution.

Cumulatively, more than 1000 days of antibiotics were administered during the study period, with no consistent effect on viral load or gastrointestinal symptoms despite evidence that norovirus cellular invasion requires bacteria.^8^

In summary, CNI in patients with CVID is associated with almost complete absence of CD27^+^ B cells (including switched memory B cells), B-cell dysfunction characterized by low expression of CD80 and CXCR5, a marked increase in numbers of transitional and CD21^−^ B cells, poor T-cell proliferation, and high PD-1 expression on CD4^+^ T cells. High numbers of CD21^−^CXCR5^−^ B cells and PD-1^+^ T cells both imply immunologic “exhaustion.” Diagnosis might be heralded by decreasing lymphocyte counts, which are often driven by iatrogenic immunosuppression (which can also precipitate relapse). The gut mucosa exhibits intraepithelial cytotoxic T-cell lymphocytosis and, with thorough assessment, invariably demonstrates villous atrophy (unlike recent reports^9^); malabsorption is universal. Cross-transmission within immunodeficiency services appears rare, but we nevertheless advocate strict infection control. Infections last years, with late relapses and high viral diversity. Available treatments are variably effective and require further research.

## Figures and Tables

**Fig E1:**
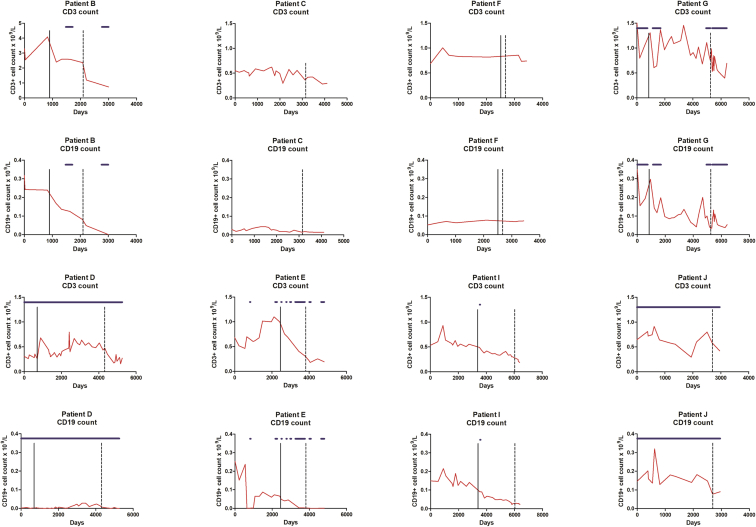


**Fig E3:**
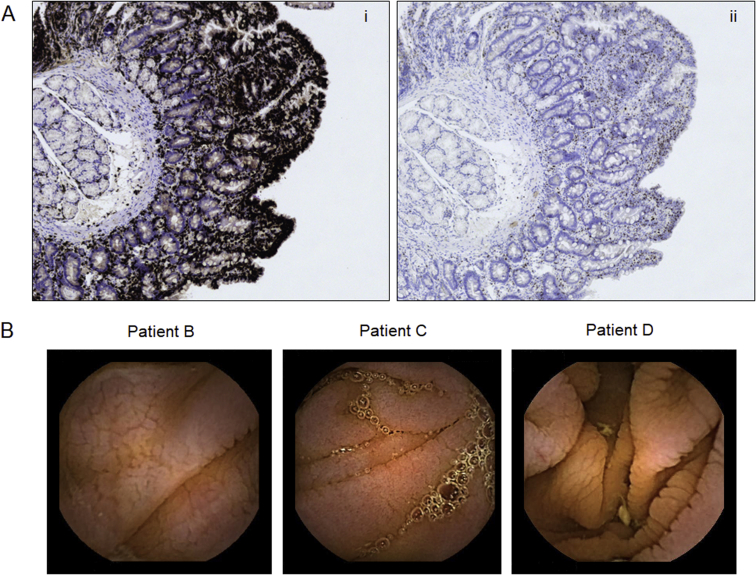


**Fig E4:**
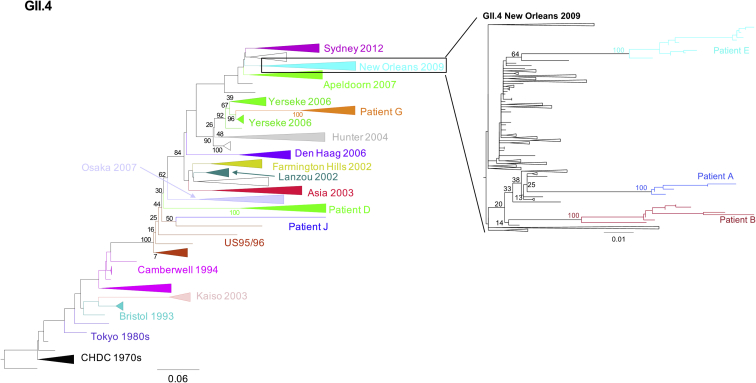


**Fig E5:**
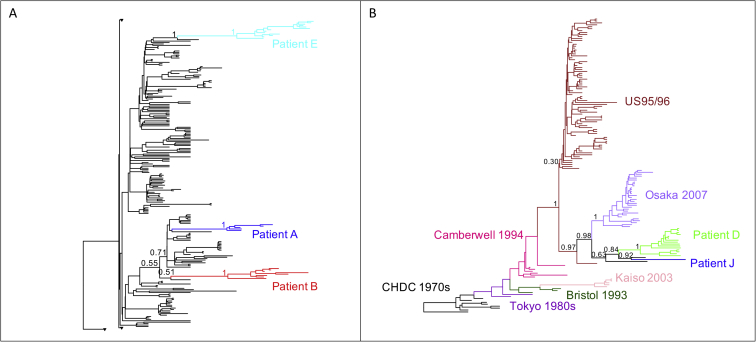


**Fig E7:**
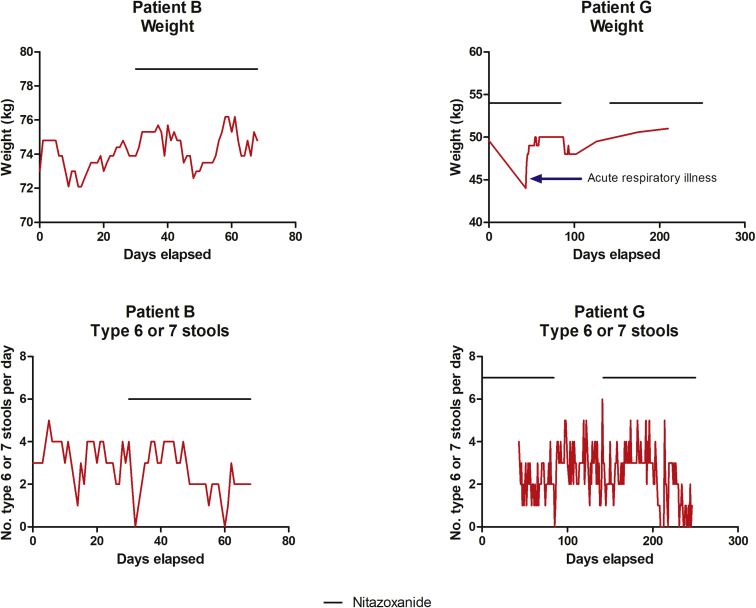


**Fig E8:**
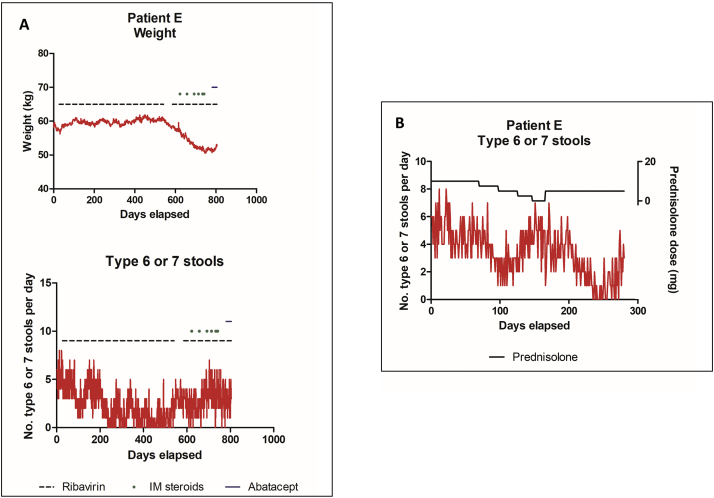


## Supplementary data

Online Repository text

Fig E2

Fig E6
