# Supplementary material for: A comprehensive characterization of chronic norovirus infection in immunodeficient hosts
Source: J Allergy Clin Immunol. 2019 Nov;144(5):1450–3. doi: 10.1016/j.jaci.2019.07.036 (PMC6843911; doi:10.1016/j.jaci.2019.07.036)
Supplement: Online Repository text [file mmc1.docx]

**Table E1.** Clinical characteristics of cohort with chronic norovirus infection

| ID | Underlying diagnosis | Significant immune suppression prior to norovirus diagnosis | Duration of norovirus infection to date (since diagnosis) | Age at diagnosis of norovirus | Co-morbidities | Serum vitamin E nadir (µmol/L) [NR 11.6-46.4] | Serum folate nadir (µg/L) [NR as indicated] | Serum vitamin B12 nadir (ng/L) [NR 160-925] |
| --- | --- | --- | --- | --- | --- | --- | --- | --- |
| A | CLL | Nil | 68 months | 51 | - Bronchiectasis - Previous Pneumocystis jirovecii pneumonia - Chronic isolation of rhinovirus - Sinusitis | 13.1 | 4.3 (3.8-16) | 490 |
| B | CVID (Freiburg 1A) | High-dose steroids | 65 months including several remission periods (longest remission 6 months) | 52 | - Autoimmune haemolytic anaemia - Liver nodular regenerative hyperplasia with portal hypertension - Inflammatory lung disease - Splenomegaly - Intermittent lymphadenopathy | **6.2** | **2.5** (3.8-16) | 308 |
| C | CVID (Freiburg 1A) | Nil | 20 months, until clearance | 38 | - Bronchiectasis - Sinusitis - Chronic isolation of rhinovirus - Previous immune thrombocytopenic purpura (ITP) - Splenomegaly | **5.9** | **3.4** (4.6-18.7) | 291 |
| D | CVID (Freiburg 1A) | Prednisolone  Infliximab | 37 months, until death | 45 | - Bronchiectasis - Pulmonary *Mycobacterium avium* infection - Rhinosinusitis - Splenomegaly - Granulomatous liver disease - Peyronie’s disease | **3.1** | **3.1** (3.8-16) | 299 |
| E | CVID (Freiburg 1B, later 1A) | Prednisolone Rituximab | 47 months, until death,  including remission for 11 months | 53 | - Sinusitis - Chronic isolation of rhinovirus - Autoimmune haemolytic anaemia - Previous ITP - Autoimmune neutropenia - Liver nodular regenerative hyperplasia - Cervical spondylosis - Inflammatory lung disease - Previous marked splenomegaly | 17.9 | 4.4 (3.8-16) | **134** |
| F | CVID (Freiburg 1B, later 1A) | Nil | 48 months, Including remission for 14 months | 38 | - Bronchial dilatation - Splenomegaly | **10.8** | 7.2 (4.6-18.7) | 476 |
| G | CVID (Freiburg 1A) | High-dose steroids | 47 months | 50 | - Inflammatory / granulomatous lung disease - Splenomegaly - Granulomatous liver disease - Granulomatous adenitis - Osteoporosis | 13.8 | **3.2** (3.8-16) | 461 |
| H | CVID (Freiburg 1A) | MMF  Prednisolone | 31 months | 52 | - Inflammatory lung disease - Barrett’s oesophagus - Splenomegaly | 22.7 | 3.1 (2.9-50) | 371 |
| I | CVID (Freiburg 1B, later 1A) | Prednisolone  Budesonide | 17 months | 42 | - Juvenile chronic arthritis - Pernicious anaemia - Previous recurrent Campylobacter infection - Splenomegaly - Inflammatory lung disease | **10.9** | **<2** (2.9-50) | 446 |
| J | CVID (Freiburg 1A) | Prednisolone | 24 months | 47 | - Liver nodular regenerative hyperplasia with portal hypertension - Splenomegaly - Bronchiectasis - Thrombocytopenia | **<1.4** | **4.1** (4.6-18.7) | 335 |

MMF, mycophenolate mofetil

**Table E2**. Gastrointestinal findings in patients with chronic norovirus infection.

| ID | Radiological findings | Small bowel endoscopic findings | Histology | Immunohistochemistry  (0 = absent, + = present. ++ to +++++ = increased mildly to massively) | |
| --- | --- | --- | --- | --- | --- |
| A | CT enterography 19/08/13: Distended stomach, normal appearance of small bowel. | OGD 29/08/13: No obvious pathology  Capsule endoscopy 26/11/12: severe villous atrophy in proximal and mid small bowel. Very proximal duodenum slightly spared | Duodenal mucosa: normal. | CD3: ++  CD4: +  CD8: ++  CD19: 0  Granzyme: ++  Perforin: 0/+ | |
| B | Not performed | OGD 01/05/14: Normal duodenum  Capsule endoscopy: 22/09/14: Severe villous atrophy of entire small intestine, mucosal oedema, mosaicism of the mucosa, notching of small intestinal folds | Duodenum: Villous blunting and increased intraepithelial lymphocytes | CD3: ++++  CD4: ++  CD8: ++++  CD19: 0  Granzyme: ++++  Perforin: + | |
| C | Small bowel MRI 03/10/14: mild non-specific fold thickening and distension in the jejunum. | OGD 31/07/14: Normal  Capsule endoscopy 18/06/15: profound villous atrophy and mucosal oedema noted throughout the entire small bowel. | Duodenum: Normal | CD3: +  CD4: +  CD8: +  CD19: 0  Granzyme: +  Perforin: 0/+ | |
| D | Not performed | Capsule endoscopy 05/10/09: proximal small bowel has villous blunting and notched appearance, mucosa has a mosaic appearance.  OGD 13/11/14: Geographical appearance of the duodenal mucosa with flattened villi | Duodenum: significant villous blunting and intraepithelial lymphocytosis | CD3: +++++  CD4: ++++  CD8: +++++  CD19: 0  Granzyme: +++++  Perforin: +++ | |
| E | CT enterography 13/12/12: normal | OGD 06/09/12: normal duodenum  Double balloon enteroscopy 29/07/13:  Duodenal flattened villi, remainder of small bowel unremarkable | Duodenum, ileum, jejunum: Mild villous atrophy and increased intraepithelial lymphocytes | Duodenum:  CD3: ++  CD4: +  CD8: ++  CD19: 0  Granzyme: +  Perforin: 0/+ | Ileum:  CD3: +++++  CD4: ++++  CD8: +++++  CD19: 0  Granzyme: +++  Perforin: +++ |
| F | Not performed | OGD 28/09/15: normal appearances | Duodenum: blunted villi, crypt hyperplasia and increased intraepithelial lymphocytes | CD3: +++  CD4: +++  CD8: +++  CD19: 0  Granzyme: ++  Perforin: + | |
| G | CT enterography 27/01/16:  Normal | Not performed | N/A | N/A | |
| H | CT enterography 28/07/18:  Thickening of 3^rd^/4^th^ part of duodenum and proximal jejunum with adjacent small mesenteric nodes. | OGD 2/08/18: mild gastritis, otherwise normal mucosa | Duodenum: Patchy flattening of villi, increased intra-epithelial lymphocytes | Not performed | |
| I | CT enterography 06/05/18:  Mild wall thickening of the distal colon. | OGD 12/05/18: normal upper GI tract  Capsule 01/06/15: mild terminal ileal intestinal nodular lymphoid hyperplasia | Duodenum: Moderate to severe villous blunting, crypt hyperplasia, intraepithelial lymphocytosis | Not performed | |
| J | Not performed | OGD 10/11/17: duodenal scalloped mucosa with flattened villi. | Duodenum: mucosal denudation, villous shortening, increased inflammatory infiltrate. | Not performed | |

**Table E3**. Estimating infection interval for each patient.

| **Patient** | **Infecting genotype/variant** | **Years in which GII.4 variant circulated** | **Divergence date** | **Ancestor date** | **Date of first positive sample** | **Earliest NGS sample** | **Infection interval** |
| --- | --- | --- | --- | --- | --- | --- | --- |
| A | GII.P4 New Orleans 2009/GII.4 New Orleans 2009 | 2009-2012 | Mar-2011 | Aug-2013 | Oct-2013 | Nov-2013 | Mar-2011 – Aug-2013 |
| B | GII.P4 New Orleans 2009/GII.4 New Orleans 2009 | 2009-2012 | Nov-2010 | Sep-2013 | Feb-2014 | Dec-2014 | Nov-2010 – Sep-2013 |
| C | GII.P7/GII.14 (re-infected with GII.P16/GII.4 Sydney 2012) | NA | Sep-2004 | Dec-2014 | Jun-2014 | Dec-2014 | Sep-2004 – Jun-2014 |
| D | GII Could not assign/GII.4 Could not assign | NA^a^ | Sep-2004 | NE^c^ | Jul-2014 | Dec-2014 | Sep-2004 – Jul-2014 |
| E | GII.P4 New Orleans 2009/GII.4 New Orleans 2009 | 2009-2012 | Nov-2010 | Apr-2014 | Jul-2014 | Dec-2014 | Nov-2010 – Apr-2014 |
| F | GII.P17/GII.17 | NA | Dec-2014 | NE^d^ | May-2015 | Aug-2015 | Dec-2014 – May-2015 |
| G | GII.P4 Could not assign/GII.4 Yerseke 2006a | 2006-2009 | After Jul-2003^b^ | NE^c^ | Jul-2015 | Sep-2015 | Jul-2003 – Jul-2015 |
| I | GII.P7/GII.6 | NA | Nov-2010 | NE^d^ | Jan-2018 | May-2018 | Nov-2010 – Jan-2018 |
| J | GII.P4 Could not assign/GII.4 could not assign | NA^a^ | Jun-2007 | NE^d^ | Jun-2017 | May-2018 | Jun-2007 – May-2018 |

Sample consensus sequences were genotyped using the norovirus genotyping tool. Divergence dates were calculated as the median date of divergence of the patient virus sequences from the remaining sequences in the corresponding genotype or variant.

^a^ The viruses from patients D and J were genotyped as GII.4 but could not be genotyped to the variant level

^b^ Estimated from the ancestor date of the GII.4 Yerseke 2006a variant

^c^ Not estimated due to lack of temporal signal

^d^ A single sample was sequenced from each of these patients

**Table E4.** T cell proliferation results.

| Patient ID | Underlying diagnosis | On immunosuppression at time of testing | T cell proliferation with PHA stimulation (percentage of contemporaneous control) |
| --- | --- | --- | --- |
| A | CLL | No | 7.0% |
| B | CVID | Yes | 1.1% |
| C | CVID | No | 12.0% |
| D | CVID | Yes | 1.2% |
| E | CVID | Yes | 0.5% |
| F | CVID | No | 24.9% |
| G | CVID | Yes | 11.5% |
| H | CVID | Yes | 9.1% |
| I | CVID | No | 4.3% |
| J | CVID | Yes | 33.7% |

**Supplementary Methods: Control patient recruitment and ethical approvals**

Control patients for immunological tests were selected among other Common Variable Immunodeficiency (CVID) patients managed in the clinical service or from healthy individuals. All CVID patients and healthy controls provided written informed consent to provide samples (and symptom diaries where required) for research purposes with approval from the NRES Committee London (REC 04/Q0501/119 and REC 08/H0720/46 respectively). Control samples for measures of viral diversity were selected from two sources. The first were 31 stool samples from adults (median age 81 years, age range 20–94 years) testing positive for norovirus by PCR during an in-patient norovirus outbreak at Norfolk and Norwich University Hospital (NNUH); 9/31 patients were immunosuppressed. Duration of infection is not known but all had acute norovirus infections associated with the outbreak, with specimens collected soon after the onset of symptoms. The second were 49 stool samples from children (median age 2 years, age range 2 months –16 years) at Great Ormond Street Hospital for Children (GOSH); 30/49 patients were immunosuppressed. All of the specimens from NNUH and GOSH were anonymised residual diagnostic specimens collected soon after the onset of symptoms; sequencing of these specimens was approved by NRES Committee London (REC 14/LO/1331).

**Figure Legends**

**Figure E1.** CD3+ T cell and CD19+ B cell counts over time in CVID patients with chronic norovirus infection. Dotted vertical line represents date norovirus was first identified. Solid vertical line represents estimated earliest date of norovirus acquisition based on virological sequencing analysis (for patients C and J this date precedes the earliest date on the x axis). Blue bars indicate significant iatrogenic immunosuppression (prednisolone >7.5mg per day, budesonide >3mg per day, and/or Depo-Medrone, infliximab, rituximab, abatacept or mycophenolate mofetil).

**Figure E2.** **A.** Flow cytometric analysis of peripheral blood mononuclear cells from patients with common variable immunodeficiency (CVID) and chronic norovirus infection (‘Norovirus patients’) and healthy controls collected and processed contemporaneously. **i-vi.** Percentages of CD19-positive B cells which were CD27-positive, switched memory cells, CD80-positive, CXCR5-positive, CD21-negative and transitional B cells. Lines and error bars represent means and standard deviation, p values from t tests. n=7 norovirus patients (one excluded due to absent CD19+ B cells), n=6 controls. * p<0.05, ** p<0.01, *** p<0.001. **vii-ix.** T follicular helper cell percentage (Tfh, defined as CXCR5-positive & PD1-positive), naïve T cell percentage (CD45RA+) and total PD1-positive percentage of CD4+ T cells. Lines and error bars represent means and standard deviation, p values from t tests. n=8 norovirus patients, n=6 controls. * p<0.05, ** p<0.01. **B.** Flow cytometric analysis of peripheral blood mononuclear cells from CVID patients without norovirus infection (‘other CVID’) and healthy controls collected and processed contemporaneously. **i-vi**. Percentages of CD19-positive B cells which were CD27-positive, switched memory cells, CD80-positive, CXCR5-positive, CD21-negative and transitional B cells. Lines and error bars represent means and standard deviation, p values from t tests. n=8 CVID patients, n=6 controls.

**Figure E3**. Mucosal appearances in chronic norovirus infection. **A**. Immunohistochemistry on duodenal biopsy from a patient with chronic norovirus infection staining for CD8 (A) and granzyme (B). Note profound villous atrophy as well as intense CD8+ intraepithelial lymphocytosis with significant granzyme expression. **B**. Endoscopic appearances of small bowel mucosa on capsule endoscopy, demonstrating villous atrophy and mucosal oedema.

**Figure E4.** Maximum likelihood phylogenetic tree containing available GII.4 VP1 sequences (n=2198) and VP1 sequences from each of the six patients infected with GII.4 viruses. GII.4 variants and monophyletic patient clades are shown in different colours. Clades have been collapsed for clarity. Bootstrap supports are shown on nodes that are important for patient clustering.

**Figure E5.** **A.** Maximum clade credibility tree of GII.4 New Orleans 2009 VP1 sequences including sequences from patients A, B and E. Posterior supports are shown on important nodes for patient clustering. Triangles represent clades that have been collapsed for clarity. **B.** Maximum clade credibility tree reconstructed on VP1 sequences from the early GII.4 lineage including sequences from patients D and J. Posterior supports are shown on important nodes for patient clustering.

**Figure E6.** **A.** Norovirus nucleotide diversity (average number of nucleotide differences between reads at a specific site) for children from Great Ormond Street Hospital for Children (Children controls), adults from Norfolk and Norwich University Hospital (Adult controls) with acute epidemic norovirus infection (one outlying point represents reinfection of the same individual) and Royal Free London patients with chronic norovirus infection (Study subjects). **B.** Diversity was calculated for each sample collected from each of six patients and is plotted here against the number of days between the collection date of the first next generation sequencing (NGS) sample and the respective sample. Samples are coloured by the patient they were collected from. **C.** As with **(B)** but here diversity is plotted against the number of years since the most recent common ancestor of the virus consensus sequences from the patient.

**Figure E7**. Improvement in weight and number of type 6 or 7 stools per day (according to the Bristol Stool Form Scale^1^) in response to nitazoxanide treatment in two patients. Type 6: fluffy pieces with ragged edges, a mushy stool; Type 7: watery, no solid pieces.

**Figure E8**. **A.** Improvement in weight and number of type 6 or 7 stools per day in response to ribavirin treatment in patient E. Ribavirin was stopped after one year of negative norovirus tests, with subsequent rapid relapse. The weight loss also coincided with development of inflammatory arthropathy requiring intramuscular (IM) steroid treatment. **B.** Patient E’s frequency of type 6 or 7 stools when on low-dose prednisolone. As the prednisolone dose was weaned down from 10mg to zero, the frequency of type 6 or 7 stools increased.

**References**

1. O'Donnell LJ, Virjee J, Heaton KW. Detection of pseudodiarrhoea by simple clinical assessment of intestinal transit rate. Bmj 1990; 300:439-40 DOI: 10.1136/bmj.300.6722.439.
