# Supplementary figures and images for: A comprehensive characterization of chronic norovirus infection in immunodeficient hosts
Source: J Allergy Clin Immunol. 2019 Nov;144(5):1450–3. doi: 10.1016/j.jaci.2019.07.036 (PMC6843911; doi:10.1016/j.jaci.2019.07.036)

## A CD19+ B cells

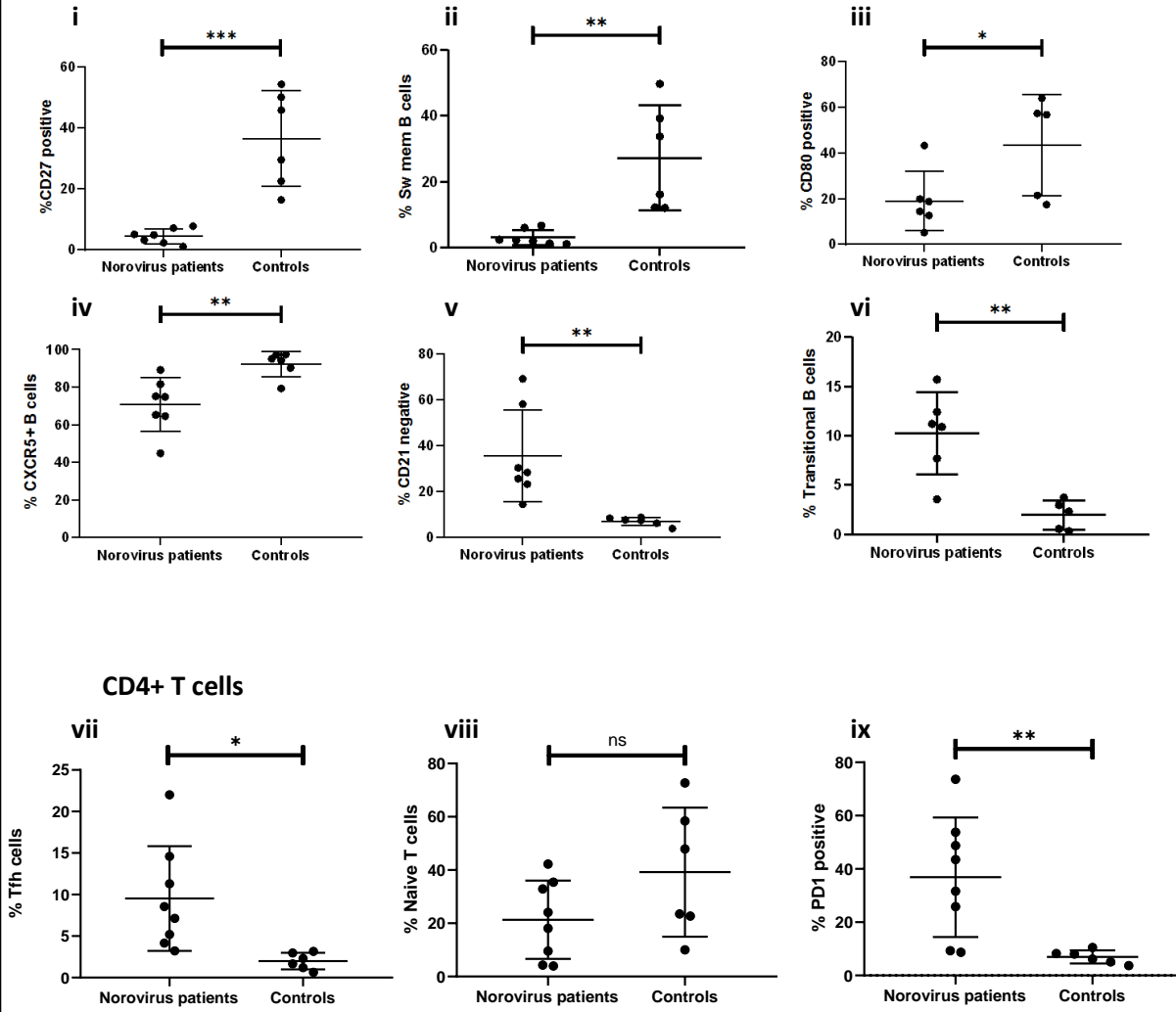

## B CD19+ B cells

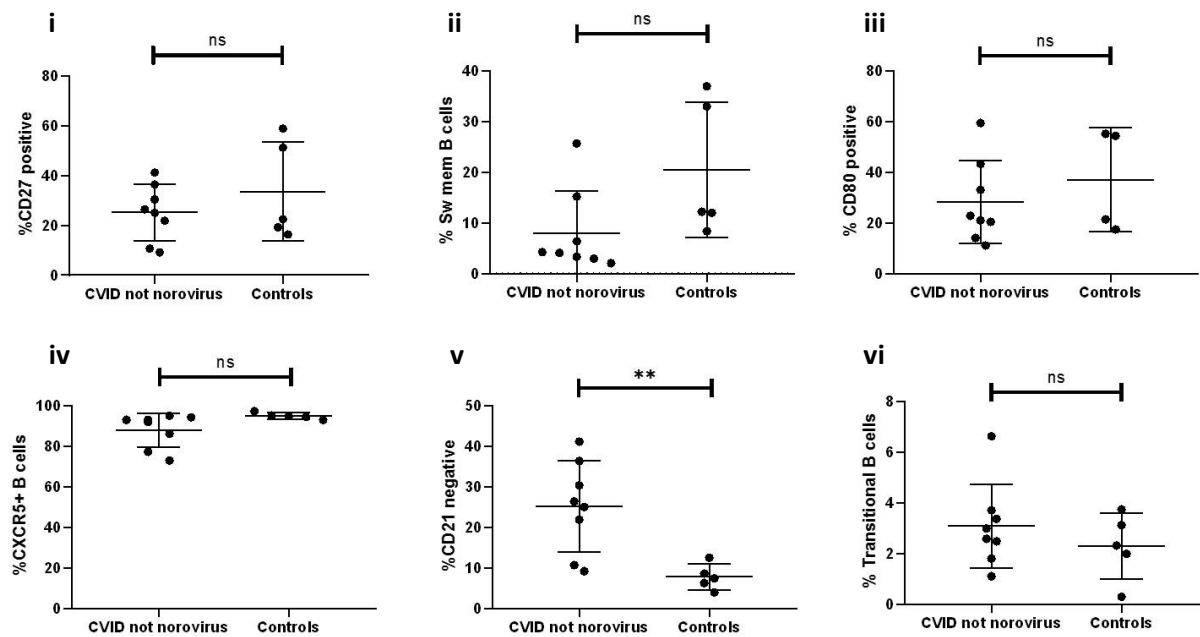

Supplement: Fig E2 [file mmc2.pdf]

**A**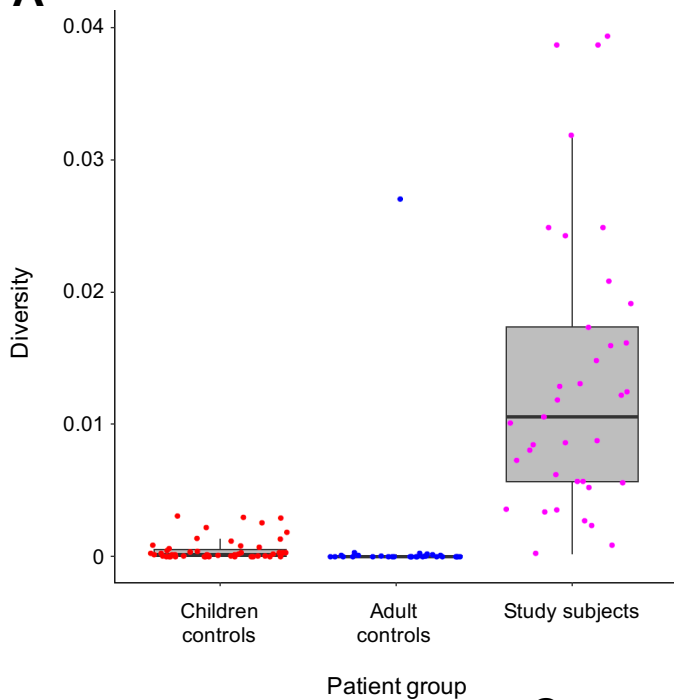**B**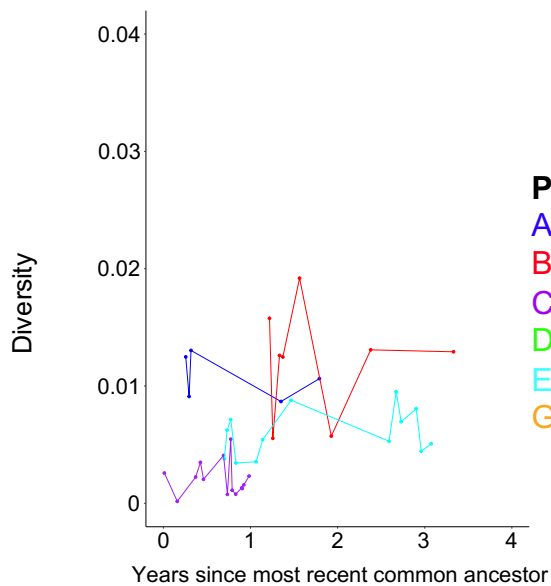**C**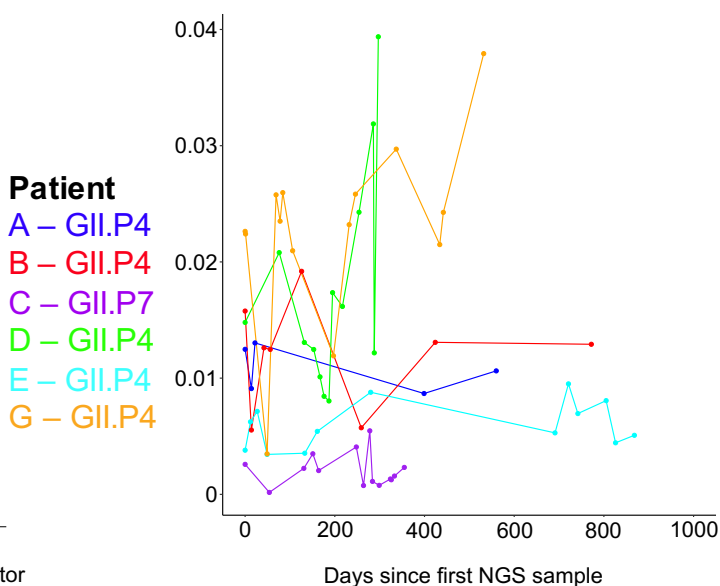

Supplement: Fig E6 [file mmc3.pdf]
